# *FgGmtB* Plays an Important Role in Growth, Reproduction, Virulence and Deoxynivalenol Biosynthesis of *Fusarium graminearum*

**DOI:** 10.3390/jof10030208

**Published:** 2024-03-11

**Authors:** Chenming Zhao, Xiaoyue Yang, Wenqiang Jiang, Guifen Zhang, Dongfang Ma

**Affiliations:** 1 Key Laboratory of Sustainable Crop Production in the Middle Reaches of the Yangtze River, College of Agriculture, Yangtze University, Jingzhou 434025, China; zhaocm121@163.com (C.Z.); yangxy_1999@163.com (X.Y.); 201872441@yangtze.edu.cn (W.J.); 2 State Key Laboratory for Biology of Plant Diseases and Insect Pests, Institute of Plant Protection, Chinese Academy of Agricultural Sciences, Beijing 100193, China

**Keywords:** GDP-mannose transporters, *Fusarium graminearum*, pathogenicity, stress responses, virulence

## Abstract

GDP-mannose transporters (GMTs) have been implicated in the virulence of some important pathogenic fungi, and guanosine diphosphate (GDP) mannose transporters transport GDP-mannose from the cytosol to the Golgi lumen prior to mannosylation, where mannose attaches to the modified protein. GMTs could be potential targets for new antifungal drugs, as disruption of any step in GDP-mannose biosynthesis can affect fungal viability, growth, or virulence. To date, the GDP-mannose transporter has been extensively studied in yeast, but its biological function in fungi, particularly *F. graminearum*, is still unclear. In this experimental study, the role of the GDP-mannose transporter in *F. graminearum* was investigated by analysing the *VRG4* gene. *FgGmtA* and *FgGmtB* were blastp-derived from their *Scvrg4* protein sequences and proved to be their functional homologues. The mutant and complementary strains of *FgGmtA*, *FgGmtB* and *FgGmtA&B* genes were generated and used to evaluate the effect of the two GMTs genes on mycelial growth, asexual reproduction, sexual reproduction, cell wall sensitivity, glyphosate synthesis and drug susceptibility. Only in the *FgGmtB* and *FgGmtA&B* mutants was the rate of mycelial growth slowed, conidium production increased, sexual reproduction impaired, cell wall sensitivity increased, glycemic content decreased, and drug sensitivity reduced. The results of the pathogenicity assessment of GMTs showed that only *FgGmtB* affects the patogenicity of *F. graminearum*. At the same time, the effect of GMTs on the ability of rhinoceros to synthesise DON toxins was investigated and the results showed that the ability of Δ*FgGmtB* and Δ*FgGmtA&B* mutants to produce the DON toxin was significantly reduced, and the expression of toxin-related genes was also reduced.

## 1. Introduction

Fusarium head blight (FHB), caused by *F. graminearum*, is an economically devastating disease of wheat and other small grain cereal crops [1,2]. *F. graminearum* produces mycotoxins such as deoxynivalenol (DON) and zearalenone in cereal grains, which further threaten human and animal health [3].

The use of triazole fungicides can effectively reduce the severity of Fusarium head blight and DON levels [4]. For example, the use of prothioconazole can reduce the incidence of the disease by 90% [5]. Recent reports indicate that overuse of the same type of fungicide has led to the emergence of resistance. Under epidemic conditions, even the most effective fungicides may not be sufficient to keep toxin levels below the critical threshold, especially in susceptible varieties [6,7]. The most effective, economical, and safe method of controlling FHB in wheat is the selection of resistant varieties. However, due to the generally poor resistance of wheat cultivars, there is currently no wheat cultivar that has been identified and has achieved complete resistance or immunity to the FHB pathogen.

GDP-mannose transport protein (GMT) belongs to a large family of nucleotide sugar transporters (NSTs) and is mainly located in the Golgi apparatus. NSTs can be subdivided into GDP sugar transporters, UDP sugar transporters and ADP sugar transporters. For instance, the protein sequences of GONST1-4 and GDP sugar transporters [8,9,10,11] in *Arabidopsis thaliana* have been shown to contain a conserved GALNK motif. In *Saccharomyces cerevisiae*, this motif has been shown to interact with the guanosine diphosphate (GDP) moiety of GDP sugars [12]. GDP sugar transporters, including *VRG4* in *Saccharomyces cerevisiae* and *GmtA* and *GmtB* in *Aspergillus nidulans*, contain conserved GALNK regions. Gao et al. [12] found that mutation of the GALNK region impaired the sugar transport activity of *VRG4*, suggesting its involvement in substrate binding. In *S. cerevisiae*, *Vrg4p* mediates GDP-mannose transport, which is a critical regulator of protein glycosylation and the first and rate-limiting step for Golgi-mediated protein mannosylation at the cell surface [13,14].

In eukaryotes, glycosylation is divided into multiple compartments to maintain efficient and precise regulation, occurring within the lumen of the endoplasmic reticulum and the Golgi apparatus. Glycosylation is a post-translational modification of certain membrane and wall components that is critical for the growth, virulence, and immunogenicity of many pathogenic organisms, including *Aspergillus* species [15,16,17,18]. Mannosylation is a type of glycosylation in which mannose polysaccharides attach to proteins or other carbohydrates to form various glycoconjugates [19]. GMT acts as a mannose donor in the steps of mannosylation, producing mannose polysaccharides and other mannose-containing glycoproteins and glycolipids. These mannose-containing glycoconjugates play a critical role in the virulence and immune response of many fungal and protozoan pathogens. Mammalian cells do not possess GDP-mannose transporters, as mannose glycosylation is limited to the utilisation of lipid-activated mannose in the endoplasmic reticulum or the conservative core glycosylation of cellular solute GDP-mannose, making GMT an excellent novel target for antifungal drugs.

Some fungal species, such as *Saccharomyces cerevisiae*, encode a single Gmt, while others, including *A. nidulans*, express two (*GmtA* and *GmtB*). In *Aspergillus*, GDP-mannose is the precursor of all mannosylation processes [20,21]. GDP-mannose is synthesised in the cytoplasm and then translocated to the Golgi lumen by the action of GDP-mannose transporters (GMTs) [22]. GMTs may be potential drug targets for new antifungal agents, as disruption of any step of GDP-mannose biosynthesis may affect fungal viability, growth, or virulence. In *A. nidulans*, for example, the Gmt-deleted strains had smaller colonies with reduced sporulation and thicker hyphal walls. The GmtA-deficient spores exhibited reduced hydrophobicity and reduced adherence and biofilm-forming ability in vitro. *GmtA* is not only involved in maintaining cell wall integrity, but also plays an important role in biofilm formation and adherence [23]. In *A. fumigatus*, inhibition of GDP-mannose pyrophosphorylase (GMPP), which catalyses the synthesis of GDP-mannose, results in a lethal phenotype including hyphal lysis, cell wall defects and impaired polarity maintenance [24]. To date, the GDP-mannose transporter has been better studied in yeast, but its biological function in fungi, particularly *F. graminearum*, is still unclear. The aim of this study is to identify and characterise the GDP-mannose transporter in fungi using reverse genetics methods and to determine its role in fungal growth, development, and virulence.

## 2. Materials and Methods

### 2.1. Strains and Plants

The wild-type strain PH-1 (NRRL 31084) of *F. graminearum* was kindly provided by Professor Huai-Gu Chen from the Wheat Disease Control and Prevention Team at the Plant Protection Research Institute, Jiangsu Academy of Agricultural Sciences. Δ*FgGmtA/B* (*GmtA/B* knockout mutant of *F. graminearum*) and Δ*FgGmtA/B-C* (*GmtA/B* complement strain) were constructed. The *Triticum aestivum* variety used in the experiment was Yangmai 158 and the *Zea mays* variety was B37.

### 2.2. Phylogenetic Tree Construction, Sequence Alignment and Conserved Motif/Domain Analysis of FgGmts

Blastp multiple sequence alignment of the wild-type *F. graminearum* strain PH-1 genome was performed in the Ensembl Fungis database (http://fungi.ensembl.org/index.html, accessed on 9 September 2021) using default parameters. Based on the alignment results, the gene with the highest homology was selected as the *Gmts* gene in wild-type PH-1, named *FgGmtA* and *FgGmtB*. The GMT sequences of *F. graminearum*, *F. pseudograminearum*, *F. oxysporum*, *Aspergillus fumigatus*, *A. nidulans*, *Neurospora crassa*, *Coccidioides immitis*, *Histoplasma capsulatum*, *Cryptococcus neoformans*. were downloaded from GenBank.

The protein domains of *FgGmtA* and *FgGmtB* were predicted using the online domain analysis software SMART (http://smart.embl-heidelberg.de, accessed on 9 September 2021) and Pfam (http://pfam.xfam.org/search, accessed on 9 September 2021) with default parameters. Multiple sequence alignments were performed using Clustal W 2.1 software for candidate protein sequences from different species and *FgGmtA/B* protein sequences from *F. graminearum*. A phylogenetic tree of GDP-mannose transporters from different species was constructed using the maximum likelihood method in MEGA 11.0 software.

### 2.3. Gene Knockout and Complementation by Protoplast Transformation

Δ*FgGmtA* and Δ*FgGmtB* gene knockout mutants are generated using the split-marker method. The 1.0 kb upstream and 1.0 kb downstream regions of *FgGmtA* and *FgGmtB* are amplified separately by overlap PCR and fused to the hygromycin phosphotransferase (hph) cassette as described in previous studies [25]. The resulting PCR products are transformed into wild-type protoplasts. Transformants resistant to hygromycin are selected by PCR using four pairs of primers: HYG/F, HYG/R, YG/F, and HY/R (Table S1).

To perform a double knockout of *FgGmtA* and *FgGmtB*, *FgGmtA* is replaced by NEO (neomycin phosphotransferase) in the *FgGmtB* single-gene knockout mutant strain. The NEO sequence is amplified from the plasmid vector pKNTG [26] using primers NEO-F1/NEO-R1 and NEO-F2/NEO-R2 (Table S1). Similarly, the homologous arm fragments LN1 and LN2 are constructed using the same method. The resulting PCR products are transformed into the protoplasts of the Δ*FgGmtB* gene knockout mutant to construct the double-gene knockout mutant (named Δ*FgGmtA&B*).

To verify the functionality of the *FgGmtA* and *FgGmtB* genes, a gene complementation experiment is required. Using PH-1 genomic DNA as a template, a gene fragment containing its own promoter and upstream stop codon is amplified using primers CF and CR (Table S1). After purification, the fragment is cloned into the pKNTG vector in a one-step cloning reaction and then transformed into DH5α-competent cells to construct the *FgGmtA/B*-GFP complementation fusion vector. The vector is then transformed into the protoplasts of the *FgGmtA/B* strain (using the same transformation method as before). G418 is used for the initial screening of transformants, followed by secondary verification using primers specific for the transformant strains. Transformants that show positive PCR results and restore the wild-type phenotype on PDA plates are selected for further experiments.

### 2.4. Determination of the Utilisation of Different Carbon Sources

All strains were activated on PDA medium plates and grown in inverted culture at a constant temperature of 25 °C for 3 days. A cake was punched with a 0.5 mm punch and placed separately in the centre of a Mineral Medium (MM) medium plate containing 30% sucrose, glucose, galactose, or mannose. After incubation at 25 °C for 3 days, the colony diameter of each strain was measured and photographed. All experiments were repeated 3 times independently.

### 2.5. Asexual and Sexual Reproduction Assays

All strains were activated on PDA medium plates and grown in inverted culture at a constant temperature of 25 °C for 3 days. Three cakes were punched with a 0.5 mm punch, cultured into Carboxymethylcellulose (CMC) liquid medium and incubated for 2 days at 25 °C. The spore suspension collected was transferred to Yeast Extract Peptone Dextrose (YEPD) liquid medium and incubated at 25 °C for 6 h. the concentration of conidia was adjusted so that there were approximately 10 conidia in each field of view observed under the microscope, and the conidial germination rate of each strain was counted by the five-point counting method using hemocytometer counting charts. The experiment was repeated three times. Spores were collected and stained with 10 μg/mL calcofluor white (CFW, Sigma, St. Louis, MO, USA) to visualise the cell wall and septa of *F. graminearum* spores. After 30 s, images were captured by fluorescence microscopy and the diameter, length, and number of septa of the spores were measured and recorded. After incubation at 25 °C for 5 days, spores were collected and counted using a haemocytometer. Microscopic images of spore conidiophores were taken after 24 and 48 h of incubation at 25 °C. All strains were cultured on Yeast Extract Mannitol Agar (YMA) medium and incubated for 6 and 12 h under a black light lamp. After incubation, mycelia were collected and RNA was extracted for quantitative analysis of the expression of sporulation-related genes.

All strains were cultured separately on Carrot (CA) medium and incubated at 25 °C for 3 days. After removal of the mycelium, 0.1% Tween 20 was added. Cultures were further incubated at 25 °C under a black light lamp to induce perithecia formation. After 2 weeks, perithecia, perithecial walls and ascospores were photographed under a microscope. All strains were individually cultured into the CA medium and incubated for 5 days at 25 °C under a black light lamp for 12 h, followed by another 12 h in the dark. Mycelia were harvested and RNA was extracted. As a control, mycelia were also collected after 5 days of incubation at 25 °C under normal light conditions. A quantitative analysis of the expression of genes related to sexual reproduction was performed.

### 2.6. The F. graminearum Pathogenicity Test

To investigate the role of the GDP-mannose transporter protein in the pathogenic mechanism of *F. graminearum*, each strain was cultured using wheat heads, corn silks, and wheat coleoptiles.

All strains were activated on PDA medium plates and grown in inverted culture at a constant temperature of 25 °C for 3 days. Three cakes were punched with a 0.5 mm punch and cultured into a CMC liquid medium and cultured at 25 °C for 3 days. The spore suspension was collected and prepared at a concentration of 1.5–2 × 10^5^ spores/mL in 10 μL. During wheat anthesis, 5 replicates of each mutant were injected into wheat heads. The injected spikes were placed in bags to maintain humidity after watering. Following a period of 2 days, the bags were removed, and spike symptoms were recorded 14 days later. Grains infected at the injection sites were collected for toxin determination.

Spore suspension was prepared at a concentration of 1.0–1.5 × 10^7^ spores/mL. Sterile cotton strips were soaked in the spore suspension. Following the removal of the top 2–3 cm of the wheat coleoptiles from 3-day-old germinated wheat embryos, the seedlings were wrapped in the cotton strips. Subsequently, the seedlings were cultivated at a temperature of 25 °C for a period of 5–6 days, after which the discoloured wheat coleoptile sheaths were photographed.

Fresh corn silks were collected and both ends were neatly trimmed. Four corn silks were tied together and placed on filter paper moistened with an adequate amount of water. A cake was punched with a 0.5 mm punch and placed it at the ends of the four corn silks. After being cultivated at 25 °C for 5 days, the discoloured corn silks were photographed.

Each strain was replicated 5 times, and all experiments were independently repeated 3 times.

### 2.7. Determination of Deoxynivalenol (DON) Toxin in F. graminearum

All strains were activated on PDA medium plates and grown in inverted culture at a constant temperature of 25 °C for 3 days.

The glass paper was cut into circular pieces 9 cm in diameter and placed in sterile ddH_2_O. After saturating the glass paper entirely, it was horizontally placed onto PDA agar. A cake was punched with a 0.5 mm punch and placed separately in the centre of the glass paper. After 3 days of incubation at 25 °C, photographs were taken and observations were recorded. Then, the glass paper was removed and cultivation continued at 25 °C for an additional 2–3 days while observing and photographing the growth of each strain penetrating through the glass paper.

The content of deoxynivalenol (DON) in infected wheat grains 14 days after inoculation was determined by extraction. Wheat heads from the wheat head inoculation experiment were collected. Individual seeds were weighed into a 15 mL centrifuge tube; a total of 4 mL acetonitrile/water (84:16) was added, vortexed and shaken for ≥4 h on a decolourising shaker; after shaking, it was vortexed again for 2 min and the sample was filtered into a new 15 mL centrifuge tube (filtered through a funnel paper filter), and then centrifuged at 4000× *g* for 5 min; the filtrate was removed and pass through the extraction column (filtrate was transferred all over the column); 2 mL of the extract was taken with an accurate pipette into a 15 mL centrifuge tube and blow-dried slowly with a nitrogen blower. A total of 2 mL of the extract was transformed into a 15 mL centrifuge tube and dried slowly with a nitrogen blower; a total of 0.5 mL of mobile phase (methanol) was added and filtered through a 0.22 μm microporous membrane (organic phase); the filtrate was transferred into a 2 mL brown vial for chromatographic determination. The DON toxin content in the inoculated wheat heads of each strain was determined. Three cakes were punched with a 0.5 mm punch and cultured into a CMC liquid medium and cultured at 25 °C for 5 days. After incubation, spore suspensions were collected and adjusted to a concentration of 1.5–2 × 10^5^ cells/mL in a TBI liquid medium. The cultures were incubated on a shaker in the dark at 25 °C and 170 rpm for 6 days, and mycelia were harvested. RNA extraction was performed, followed by quantitative analysis of gene expression related to deoxynivalenol (DON) toxin synthesis.

### 2.8. Sensitivity of Mycelial Growth to Cell Wall Disruptor Multiple Stress Detection

All strains were activated on PDA medium plates and grown in inverted culture at a constant temperature of 25 °C for 3 days. A cake was punched with a 0.5 mm punch and placed separately in the centre of a PDA medium plate containing 0.01% sodium dodecyl sulfate (SDS), 300 μg/mL Congo red (CR) and 200 μg/mL fluorescence brightener (CFW). After incubating at 25 °C for 3 days, the colony diameter of each strain was measured and photographed. All experiments were repeated 3 times independently.

### 2.9. Determination of Fungal Susceptibility to Fungicides

All strains were first activated on PDA medium plates and grown in inverted culture at a constant temperature of 25 °C for 3 days. A cake was punched with a 0.5 mm punch and placed separately in the centre of a PDA medium plate containing 0.5 μg/mL isobactilide or 0.25 μg/mL fludioxonil. After incubation at 25 °C for 3 days, the colony diameter of each strain was measured and photographed. All experiments were repeated 3 times independently.

### 2.10. Statistical Analysis

Data are presented as the mean of triplicates ± standard error of the mean, and statistical significance was determined by Duncan’s multiple range test at a significance level of *p* < 0.05 or *p* < 0.01 using SPSS Statistics 25.

## 3. Results

### 3.1. Sequence Alignment and Phylogenetic Analysis of FgGmts Proteins from Different Organisms

**Figure 1:**
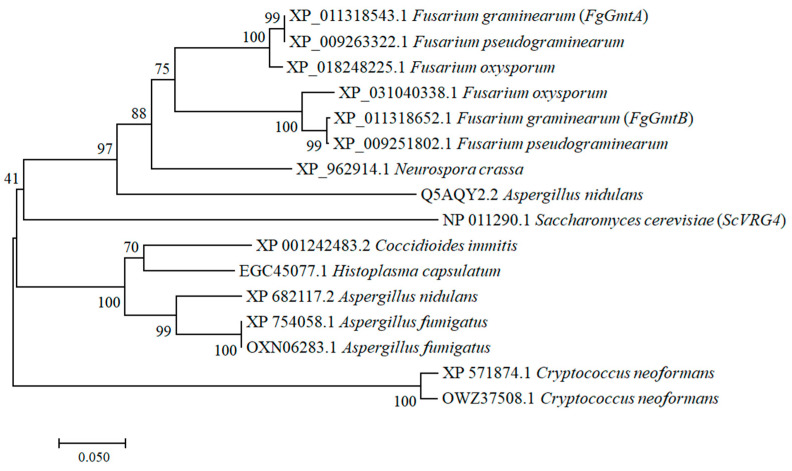
Phylogenetic tree showing the evolutionary relationships among fungal *FgGmtA* and *FgGmtB* proteins. Phylogenetic analysis of *FgGmtA* and *FgGmtB* with orthologs from other fungal species. The phylogenetic tree was constructed using MEGA 11.0 with full-length protein sequences and the neighbour-joining method with 1000 bootstrap replicates.

Using the amino acid sequence of the GDP-mannose transporter protein *ScVRG4* (YGL225W) from *Saccharomyces cerevisiae* as a reference, BLAST alignment analysis was performed using the *F. graminearum* genome database from Ensemble (http://fungi.ensembl.org/index.html, accessed on 9 September 2021). This led to the identification of homologous proteins in *F. graminearum*, namely *FgGmtA* (*FGSG_02598*) and *FgGmtB* (*FGSG_02699*). Phylogenetic analyses revealed that *FgGmtA* and *FgGmtB* homologs are present in fungi (Figure 1). In particular, we found that *FgGmtA* and *FgGmtB* orthologs are highly conserved in some species of ascomycetes. Analysis of the SMART domain showed that *FgGmtA* and *FgGmtB* contain nine and seven transmembrane domains, respectively (Figure S1).

### 3.2. Function of FgGmtA and FgGmtB in F. graminearum with S. cerevisiae VRG4 Can Complement Each Other

**Figure 2:**
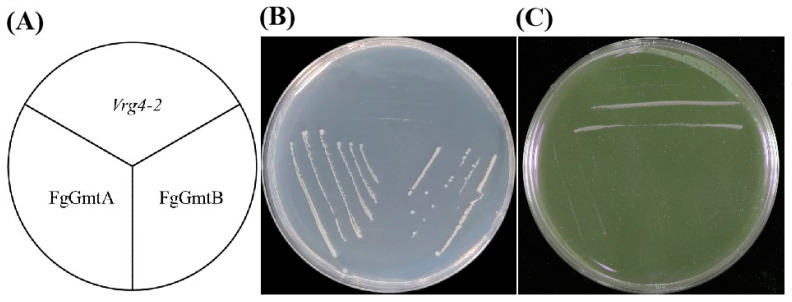
Functional complementation of *vrg4-2* yeast transformed with *FgGmts* proteins. Yeast strain *NDY5* (*vrg4-2*) was transformed with pYES2-FgGmtA and pYES2-FgGmtB. (**A**) Schematic diagram of yeast strains introduced with different target genes (pYES2-FgGmtA, pYES2-FgGmtB) and yeast strain NDY5 (*vrg4-2*) arranged on SC-Ura medium. (**B**) SC-Ura medium supplemented with 500 μg/mL hygromycin B. (**C**) SC-Ura medium supplemented with 5 mmoL/L sodium orthovanadate.

Since the *VRG4* gene in *S. cerevisiae* encodes the Golgi GDP-mannose transporter, the mutant yeast strain with a defect in GDP-mannose transport (*vrg4-2*) is unable to produce sufficient amounts of the GDP-mannose transporter. In addition, this mutant strain is sensitive to hygromycin B and shows resistance to vanadate [13]. To verify the functionality of *FgGmts* in GDP-mannose transport, yeast expression vectors pYES2-FgGmtA and pYES2-FgGmtB were constructed and introduced into the *vrg4-2* strain. The yeast strains introduced with different target genes (pYES2-FgGmtA, pYES2-FgGmtB) and yeast strain NDY5 (*vrg4-2*) were inoculated on SC-Ura medium using the scribing method according to the schematic of the lines of yeast strains shown in Figure 2A. On SC-Ura medium supplemented with hygromycin B, the pYES2-FgGmtA and pYES2-FgGmtB strains were able to grow, whereas the *NDY5* strain could not (Figure 2B). On vanadate-supplemented SC-Ura, the opposite was true (Figure 2C). On SC-Ura plates, *FgGmtA* and *FgGmtB* complemented the sensitivity of the *vrg4-2* strain to hygromycin B and the vanadate phenotype. This shows that *FgGmtA* and *FgGmtB* in *F. graminearum* complement the phenotype of the GDP-mannose transport-defective yeast mutant (*vrg4-2*), which exhibits sensitivity to vanadate and resistance to hygromycin B, indicating that *FgGmtA* and *FgGmtB* are GDP-mannose transporters.

### 3.3. FgGmts Knockout and Complementation in F. graminearum

To study the biological function of *GmtA* and *GmtB* in *F. graminearum*, Gmt gene deletion mutants were constructed by homologous recombination. PCR analysis was performed using four pairs of primers (Table S1) to identify three potential knockout mutants. The results of PCR gel electrophoresis are shown in Figure S2B–D. Sequential detection using 5F + 6R, 7F + H855R, H855F + 8R and H852 + H850 primers showed that Δ*FgGmtA* (Figure S2B) and Δ*FgGmtB* (Figure S2C) mutants were able to detect the *hygromycin B* gene but not the corresponding target gene sequence. Sequential detection using 5F + 6R, 7F + N855R, N855F + 8R, and N852 + N850 primers showed that the Δ*FgGmtA&B* mutant (Figure S2D) was able to detect the NEO gene (NEO is the gene encoding neomycin phosphotransferase) but not the *FgGmtA* and *FgGmtB* gene sequences. As a result, three mutant strains were obtained: Δ*FgGmtA*, Δ*FgGmtB* and Δ*FgGmtA&B*.

### 3.4. FgGmtB Is Involved in the Regulation of Mannose Utilisation

In *S. cerevisiae*, the loss of *VRG4* function is lethal, but some mutants retain partial transport activity. The *vrg4-2* mutation is located in a conserved region of the VRG4 protein involved in GDP-mannose binding. These mutations can be compensated for by overexpression [8,9]. In our study, we found that VRG4, *GmtA* and *GmtB* belong to the class of GDP-mannose transporters, and there is a conserved GALNK region in GDP-mannose transporters, which is contained in VRG4, *GmtA*, and *GmtB*. Gao et al. [12] found that when the GALNK region was mutated, the sugar transport capacity of VRG4 was reduced. They proposed that this region is responsible for substrate binding. We therefore hypothesised that *FgGmtA/B* is involved in GDP-mannose transport. The results showed that the growth of the Δ*FgGmtA* mutant on MM containing these four sugars was not significantly different from that on PH-1. However, the difference in growth rate between the Δ*FgGmtB* mutant and PH-1 on MM containing mannose was reduced (Figure S3). This confirms that this gene may be associated with GDP-mannose metabolism.

### 3.5. FgGmtB Is Involved in Hyphal Growth in F. graminearum

**Figure 3:**
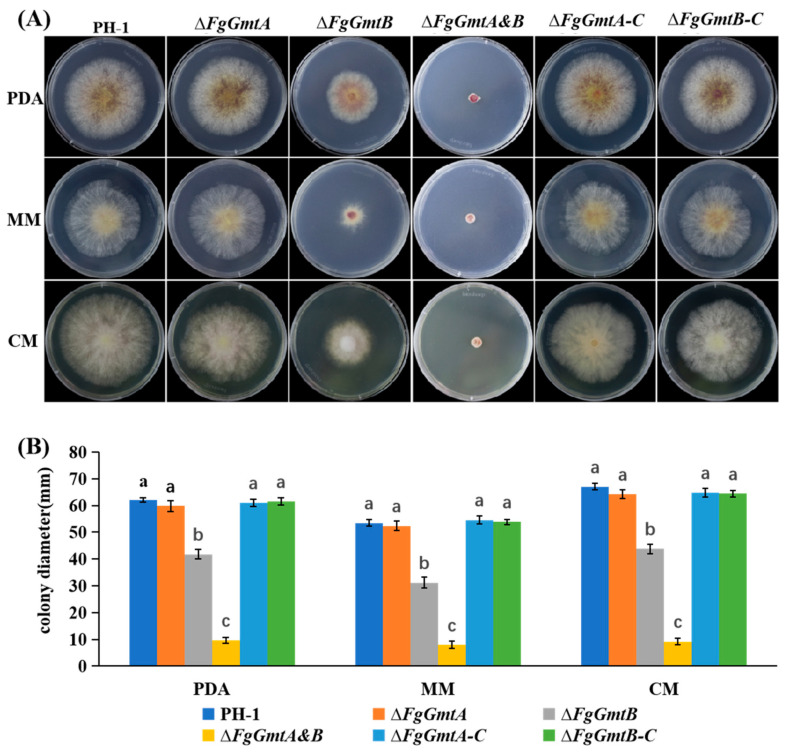
Growth of the *FgGmts* mutant on PDA, CM, and MM media. (**A**) Colonies of PH-1, Δ*FgGmtA*, Δ*FgGmtB*, Δ*FgGmtA&B*, and complemented strains were cultured on PDA, CM, and MM plates and photographed at 25 °C for 3 days. (**B**) Colony diameters of PH-1, Δ*FgGmtA*, Δ*FgGmtB*, Δ*fgGmtA&B*, and complemented strains. Values on bars followed by different letters statistically different at *p* < 0.05.

To determine the role of *FgGmtA* and *FgGmtB* in hyphal growth, the mutants were cultured into PDA medium. Compared to PH-1, the growth rates of Δ*FgGmtB* and Δ*FgGmtA&B* mutants were reduced by 38.4% and 84.6%, respectively (Figure 3B). To assess whether the growth defects of Δ*FgGmtB* and Δ*FgGmtA&B* mutants were medium-dependent, we also tested the vegetative growth of all strains on CM and MM media. It was found that both Δ*FgGmtB* and Δ*FgGmtA&B* mutants exhibited slower growth rates (Figure 3A). The complemented strain, Δ*FgGmtB-C,* showed a restoration of hyphal growth rate similar to PH-1 (Figure 3B). These results indicate that the *FgGmtA* and *FgGmtB* genes play a crucial role in the hyphal growth process of *F*. *graminearum*. Specifically, *FgGmtB* is particularly important in regulating the growth rate of hyphae.

### 3.6. FgGmtB Is Important for Asexual Reproduction

**Figure 4:**
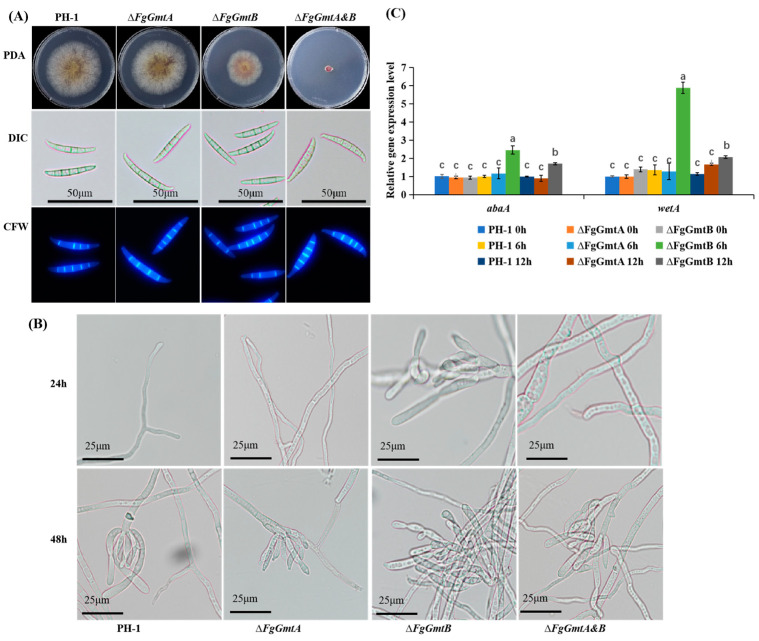
Spore-forming structure, sporulation, and sporulation gene transcription levels of PH-1, Δ*FgGmtA*, Δ*FgGmtB* and Δ*FgGmtA&B*. (**A**) The cell membranes and nuclei of PH-1 and knockout mutants were stained with CFW and observed under a microscope. (**B**) Conidia plots of PH-1 and knockout mutants. (**C**) After culturing PH-1, Δ*FgGmtA* and Δ*FgGmtB* under black light for 0, 6 and 12 h, respectively, to induce sporulation, the transcript levels of *AbaA* and *WetA* were quantified using fluorescence. Values on bars followed by different letters statistically different at *p* < 0.05.

Morphology and structure of conidia were examined under the microscope before and after staining with CFW, and no significant differences were observed between the mutant strains and the wild-type strain PH-1 in terms of conidial morphology and septal structure (Figure 4A). Compared to PH-1, both Δ*FgGmtA* and Δ*FgGmtA&B* mutant strains showed similar numbers of conidia, but the Δ*FgGmtB* mutant strain showed a significant increase (Table 1). This indicates that *FgGmtA/B* does not affect the formation of conidial cell walls and septa, but the absence of the *FgGmtB* gene can reduce conidial yield.

All strains were cultured in a CMC liquid medium for 24 or 48 h. Conidiophore images were taken under a microscope. The results are shown in Figure 3B. Conidiophores were found in Δ*FgGmtB* at 24 h, but not in the other strains until 48 h. In conclusion, *FgGmtB* affects the rate and quantity of conidia production, but has no effect on their morphology.

The *AbaA* gene encodes a protein containing ATTS DNA-binding motifs that is essential for the final stage of conidial development. *WetA* is activated by *AbaA* to complete conidiation. Since *FgGmtB* promotes sporulation, we speculated that *FgGmtB* might regulate the expression of the *AbaA* and *WetA* genes. Therefore, the expression levels of the genes *AbaA* and *WetA*, which are associated with asexual reproduction [27,28], were determined using qRT-PCR. All strains were cultured into a YMA medium. Mycelium could not be collected due to the Δ*FgGmtA&B* mutant; the expression levels of *AbaA* and *WetA* could not be determined. The results showed that under black light cultivation for 6 and 12 h compared to PH-1, the transcript levels of *AbaA* and *WetA* were significantly increased in the Δ*FgGmtB* mutant, and the increase in transcript levels was higher at 6 h than at 12 h (Figure 4C). Knockout of *FgGmtB* resulted in a significant increase in the transcript levels of *AbaA* and *WetA*. This suggests that *FgGmtB* may regulate the process of asexual reproduction by affecting the *AbaA*-*WetA* pathway, resulting in increased conidiation.

### 3.7. FgGmtB Is Important for Sexual Reproduction

**Figure 5:**
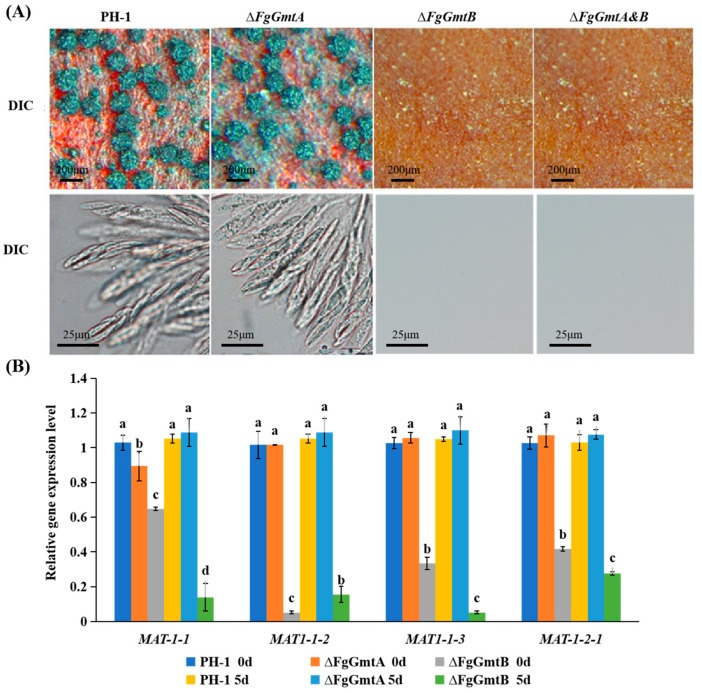
Sexual reproduction and transcript levels of genes involved in ascospore production in PH-1, Δ*FgGmtA*, Δ*FgGmtB,* and Δ*FgGmtA&B*. (**A**) Perithecium morphological maps of PH-1, Δ*FgGmtA*, Δ*FgGmtB,* and Δ*FgGmtA&B* in black light/dark (12 h:12 h) alternately after 21 days. (**B**) PH-1, Δ*FgGmtA*, Δ*FgGmtB* and Δ*FgGmtA&B* were detected in the black light/darkness treatment for 0 days and 5 days, and the transcription levels of *MAT1-1-1*, *MAT1-1-2*, *MAT1-1-3* and *MAT1-2-1* were tested. Values on bars followed by different letters statistically different at *p* < 0.05.

After 14 days of cultivation under black light induction, PH-1 and Δ*FgGmtA* produced a large number of blue-black ascocarps on the culture medium, whereas Δ*FgGmtB* and Δ*FgGmtA&B* required a longer induction time and almost no ascocarps were formed (Figure 5A). Microscopic examination of Δ*FgGmtA* ascocarps after grinding on a glass slide revealed that their morphology was similar to that of PH-1 (Figure 5A). These results showed that knockout of *FgGmtB* inhibited sexual reproduction of *F. graminearum* and prevented the formation of ascocarps.

The production and formation of ascospores is associated with many genes such as *MAT1-1-1*, *MAT1-1-2*, *MAT1-1-3,* and *MAT1-2-1* [29]. Since Δ*FgGmtB* and Δ*FgGmtA&B* mutant strains cannot form ascospores, we further investigated the transcript levels of *MAT1-1-1*, *MAT1-1-2*, *MAT1-1-3,* and *MAT1-2-1* during the sexual reproduction stage of the knockout mutants. Since Δ*FgGmtA&B* mutant strains produce almost no hyphae, they cannot be collected and the expression levels of the four genes cannot be determined. In Δ*FgGmtB*, the difference in transcript levels of the four genes compared to PH-1 increases after treatment compared to the untreated condition. However, there is no significant difference in the transcript levels of the four genes between the Δ*fgGmtA* mutant and PH-1 (Figure 5B). This indicates that *FgGmtB* upregulates the transcript levels of *MAT1-1-1*, *MAT1-1-2*, *MAT1-1-3,* and *MAT1-2-1* during sexual reproduction of *F. graminearum* and plays an important role in the regulation of sexual reproduction.

### 3.8. FgGmtB Is Involved in the Regulation of Pathogenicity of F. graminearum

**Figure 6:**
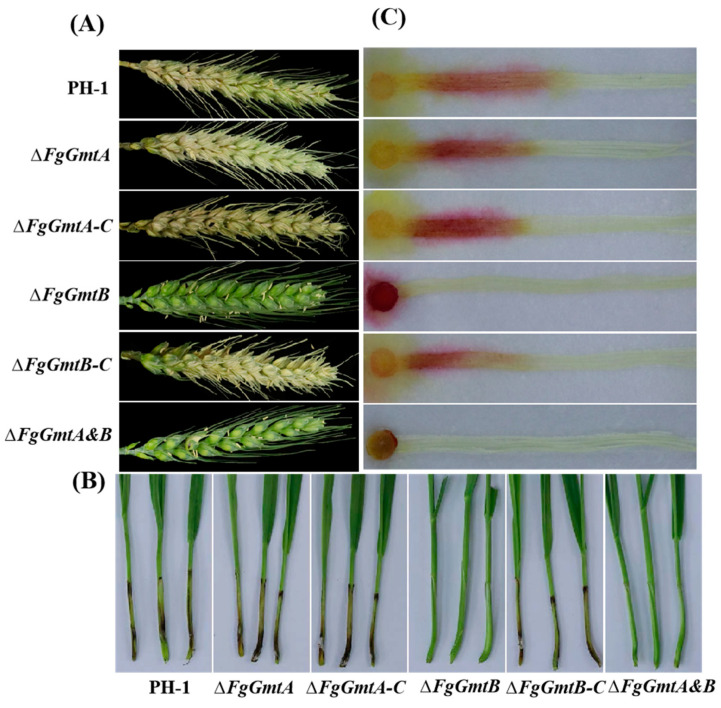
Pathogenicity tests for PH-1, Δ*FgGmtA*, Δ*FgGmtB,* and Δ*FgGmtA&B* and complementary strains. (**A**) A concentration of 1.5–2 × 10^5^ spores/mL in 10 μL was injected into wheat heads. (**B**) A concentration of 1.0–1.5 × 10^7^ spores/mL in 10 μL was injected into wheat coleoptiles. (**C**) Cakes of all strains were inoculated with corn silks.

*FgGmtB* is involved in the regulation of hyphal growth, conidiation, and perithecium formation in *F. graminearum*. To further investigate the role of *FgGmts* in the pathogenesis of *F. graminearum*, pathogenicity tests were carried out on wheat heads, wheat coleoptiles, and corn silks. The wild-type strain PH-1, Δ*FgGmtA,* and the complemented strains Δ*FgGmtA-C* and Δ*FgGmtB-C* were able to infect almost the entire wheat head, whereas Δ*FgGmtB* and Δ*FgGmtA&B* showed no symptoms and failed to infect the inoculation site (Figure 6A). Infection experiments on wheat heads, wheat coleoptiles, and corn silks showed similar results: Δ*FgGmtB* and Δ*FgGmtA&B* lost their pathogenicity, whereas the pathogenicity of PH-1, Δ*FgGmtA,* and complemented strains was essentially the same (Figure 6A–C). These results indicate that *FgGmtB* plays a crucial role in the pathogenicity of *F. graminearum* and also suggest a functional differentiation between *FgGmtA* and *FgGmtB*.

### 3.9. FgGmtB Affects DON Biosynthesis and the Expression of TRI Gene in F. graminearum

**Figure 7:**
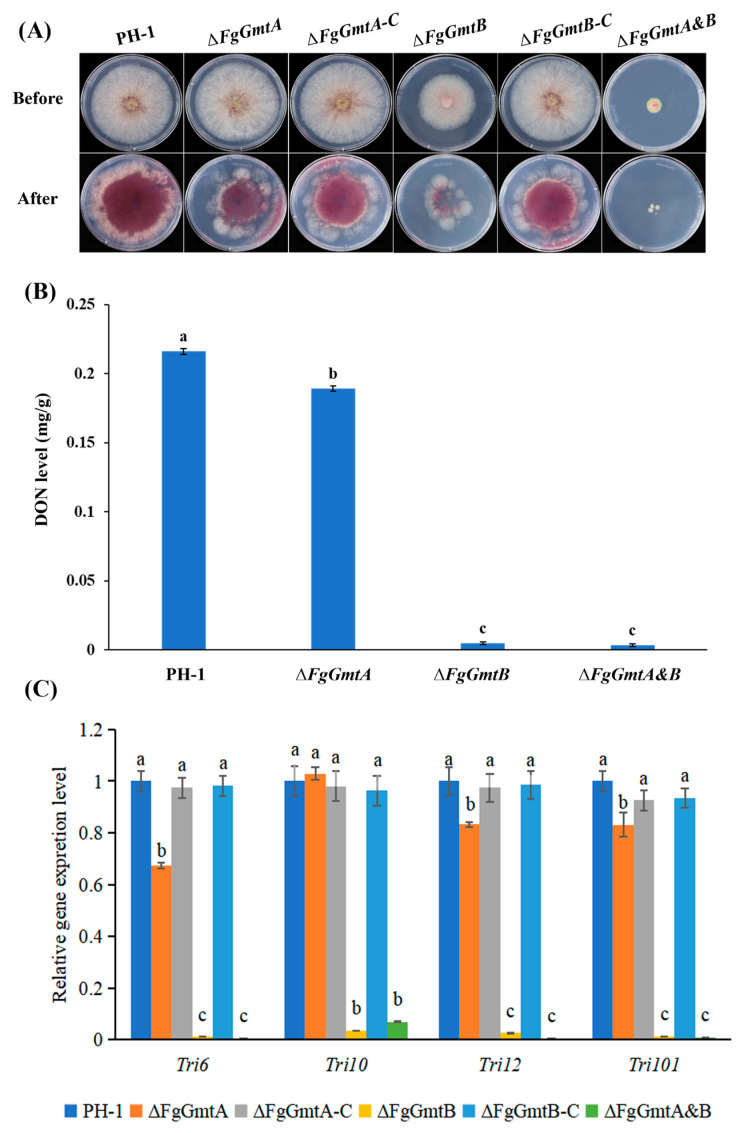
DON production and *TRI* gene expression assays of the gene knockout mutants. (**A**) Cellophane penetration experiments. (**B**) DON concentration in single grains of infected wheat at the inoculum of each strain tested after 14 days of inoculation. (**C**) Expression levels of the *TRI6*, *TRI10*, *TRI12*, and *TRI101* genes in PH-1, Δ*FgGmtA*, Δ*FgGmtA-C*, Δ*FgGmtB*, Δ*FgGmtB-C*, and Δ*FgGmtA&B*. The relative expression level of each gene in PH-1 arbitrarily set to 1. The means and standard errors calculated using data from three independent biological replicates. Means and SDs calculated from 3 independent experiments. Values on bars followed by different letters statistically different at *p* < 0.05.

The trichothecene mycotoxin deoxynivalenol (DON) produced by *F. graminearum* serves as a critical virulence factor in *F. graminearum* [30,31,32]. Plant infection assays showed that knockout of *FgGmtB* reduced the virulence of *F. graminearum*. In addition, the ability of the mutant strains to penetrate cellophane was not significantly affected in cellophane penetration experiments (Figure 7A). The results showed that the reduction in pathogenicity of the Δ*FgGmtB* strain was not caused by the regulation of hyphal penetration ability. We therefore determined the ability of Δ*FgGmtB* and Δ*FgGmtA&B* to produce DON. Compared to the PH-1, the ability of the knockout mutants Δ*FgGmtB* and Δ*FgGmtA&B* to produce DON was severely impaired (Figure 7B). To further confirm this result, the expression levels of *TRI*, which are crucial trichothecene biosynthesis genes, were confirmed by quantitative real-time PCR. The expression levels of the *TRI* gene in Δ*FgGmtB* and Δ*FgGmtA&B* were significantly decreased compared to the wild-type strain PH-1 (Figure 7C). These results indicated that *FgGmtB* plays an important role in the regulation of *TRI* gene expression and DON biosynthesis in *F. graminearum*.

### 3.10. FgGmtB Is Involved in the Regulation of Cell Wall Integrity in F. graminearum

**Figure 8:**
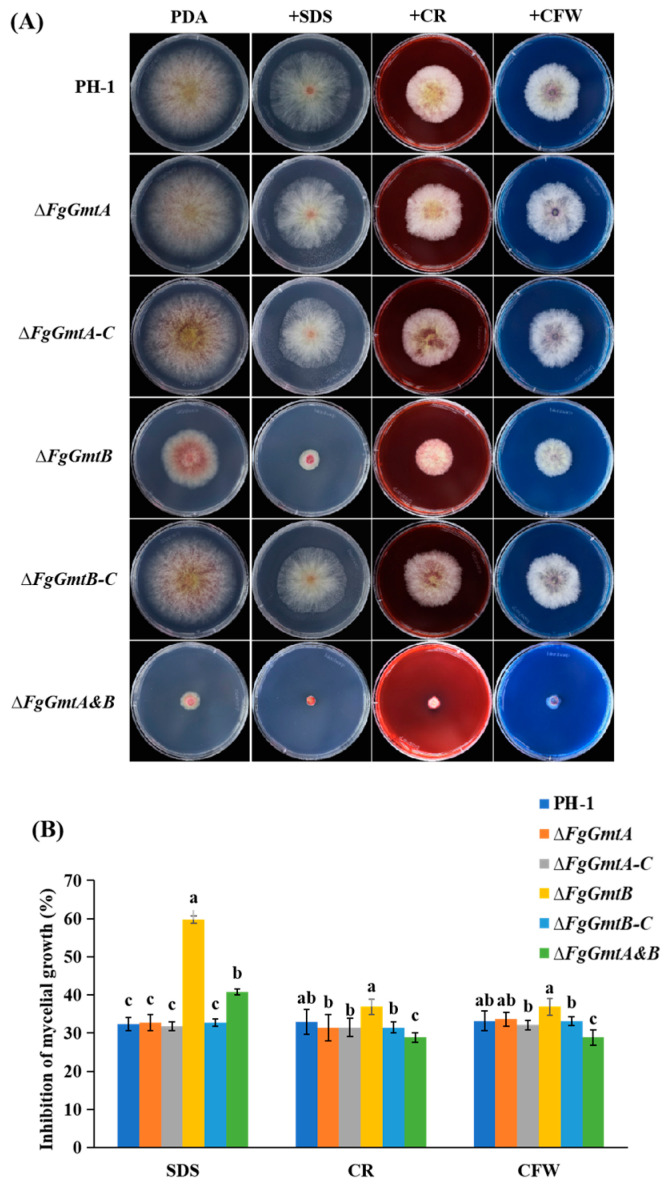
Sensitivity of Δ*FgGmtA*, Δ*FgGmtB* and Δ*FgGmtA&B* to cell wall damaging agents. (**A**) Colonies were cultured for 3 days at 25 °C on CM plates supplemented with different stress agents at indicated concentrations. (**B**) Percentage inhibition of PH-1, mutant and complemented strains. Values on bars followed by different letters statistically different at *p* < 0.05.

The fungal cell wall is the outermost and first layer of fungi and is critical for sensing the environment. The integrity of the cell wall is essential for hyphal growth and virulence. The cell wall contains substances such as chitin, cellulose, and proteins. Sodium dodecyl sulphate (SDS) is a protein denaturant that can cause protein denaturation. Congo red (CR) can bind to β-1,4-glucan in cellulose and calcofluor white (CFW) can bind to chitin. All three can affect the structure, integrity, and function of the cell wall [33,34,35]. To further investigate the role of the GDP-mannose transporter in the cell wall integrity signalling pathway, the sensitivity of all strains to cell wall disruptors such as SDS, CR, and CFW was examined (Figure 8A).

It was found that the inhibition rate of the *FgGmtB* and Δ*FgGmtA&B* mutants was significantly higher than that of the wild-type strain PH-1, while the Δ*FgGmtA* mutant strain was similar to PH-1 and showed no significant sensitivity to the above three reagents. The complemented strain essentially restored the level of the wild-type strain (Figure 8B). The Δ*FgGmtB* mutant was more sensitive to cell wall disrupting factors, and the results suggest that *FgGmtB* may be involved in cell wall formation.

### 3.11. FgGmtB Is Involved in the Regulation of Osmotic Stress and Glycerol Synthesis in F. graminearum

The virulence of *F. graminearum* is impaired by knockout of genes involved in various stress responses, including osmotic and cell wall-related [36,37]. Osmotic stress tolerance is critical for plant infection. To investigate the role of *FgGmts* in osmotic stress, all strains were cultured on CM agar plates containing 1.2 mol/L NaCl and KCl, and their growth inhibition was measured after 3 days of cultivation. The results showed that the growth inhibition rates of Δ*FgGmtB* and Δ*FgGmtA&B* mutants were more pronounced, indicating the important function of *FgGmtB* in osmotic stress (Figure S4). In addition, NaCl showed higher growth inhibition rates compared to KCl for all strains.

The hyperosmotic signalling (HOG) pathway is an important pathway by which cells respond to external osmotic stimuli. Under osmotic stress, MAP kinase kinase (MAPKK) Pbs2 phosphorylates and activates the key protein Hog1 of the HOG pathway, leading to the up-regulation of glycerol synthesis genes [38]. When fungi are subjected to external osmotic stress, the intracellular balance between osmotic pressure inside and outside the cell is maintained by the accumulation of glycerol [39,40]. Thus, the intracellular glycerol content can be used to indicate the degree of osmotic stress to which mycelial cells are subjected. Since the results of the osmotic stress experiment showed that all strains were highly sensitive to NaCl compared to KCl, to experimentally test the above hypothesis, it was only necessary to measure the glycerol content of PH-1, Δ*FgGmtB,* and Δ*FgGmtA&B* in a YEPD liquid medium with or without 1.2 mol/L NaCl. The results were as expected. Irrespective of treatment, the glycerol content in Δ*FgGmtB* and Δ*FgGmtA&B* was lower than in wild-type PH-1. Under untreated conditions, there was a significant difference in glycerol content between Δ*FgGmtB*, Δ*FgGmtA&B,* and PH-1. However, after treatment, the difference in glycerol content between Δ*FgGmtB*, Δ*FgGmtA&B,* and PH-1 decreased (Figure S4). This suggests that under salt stress, the Δ*FgGmtB* mutant accumulates more glycerol to alleviate the external stress. To summarise, *FgGmtB* plays an important role in glycerol synthesis, which in turn regulates the osmotic stress responses of *F. graminearum*.

### 3.12. FgGmtB Is Involved in Regulating the Sensitivity of F. graminearum to Fungicides

Fludioxonil, derived from the antibiotic pyrrolnitrin, is a benzimidazole fungicide, while iprodione is a dicarboximide fungicide [41]. Both fungicides are broad-spectrum and are used to control several important plant pathogenic fungi [42,43,44]. To test the sensitivity of *FgGmtA/B* to these two fungicides, all strains were cultured onto PDA plates containing isoxuron and fludioxonil and cultured for 3 days before growth inhibition was measured.

**Figure 9:**
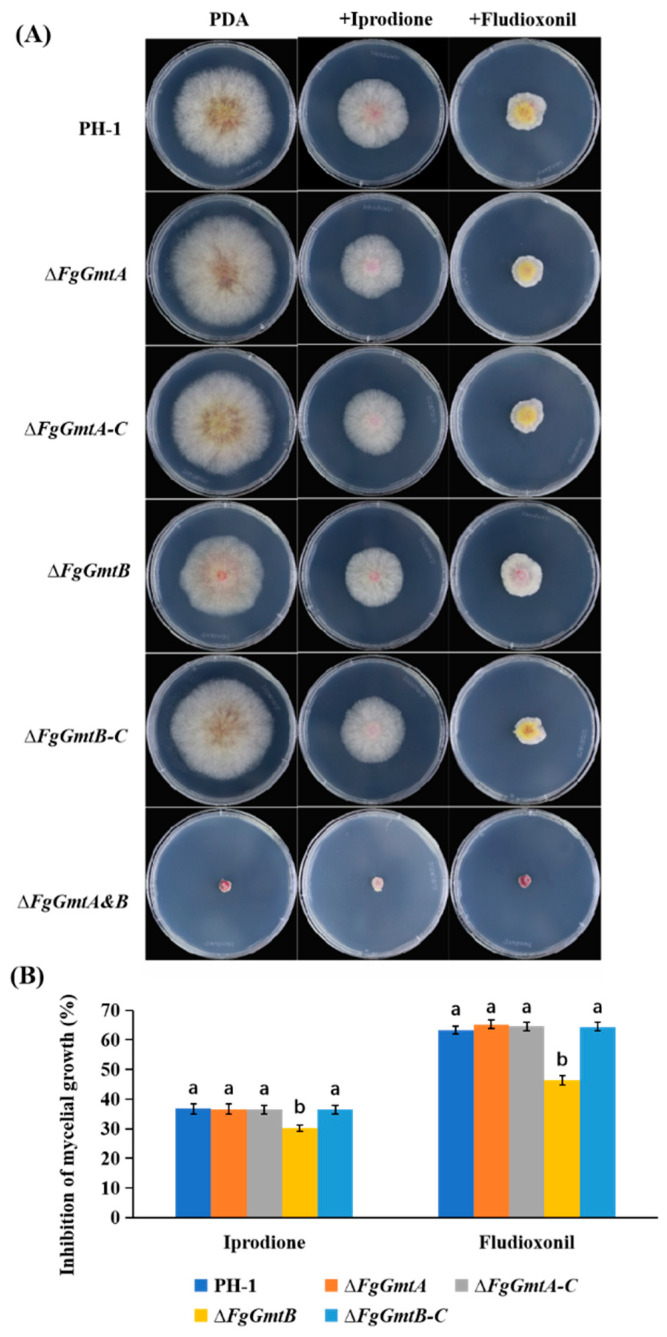
Determination of fungicide sensitivity in Δ*FgGmtA*, Δ*FgGmtB,* and Δ*FgGmtA&B*. (**A**) Colony morphology of each strain under different fungicides. (**B**) The growth of wild-type PH-1 strain, Δ*FgGmtA*, Δ*FgGmtB,* and complemented strain of the inhibition rate of each strain under different agents. Values on bars followed by different letters statistically different at *p* < 0.05.

Both fungicides inhibited the growth of the strains, with fludioxonil showing a higher inhibition rate than isoxuron. Compared to the PH-1 control, the Δ*FgGmtA* mutant showed no significant changes, whereas the inhibition rate of the Δ*FgGmtB* mutant was significantly reduced. The Δ*FgGmtA&B* strains showed no apparent growth on any of the three media, so no comparison of growth inhibition rates was performed (Figure 9). These results suggest that the *FgGmtB* gene may increase the sensitivity of *F. graminearum* to fungicides.

## 4. Discussion

In the *S. cerevisiae*, proteins and lipids are modified in the Golgi mainly by the addition of mannose, using GDP-mannose as a substrate [45]. The VRG4 gene encodes the yeast GDP-mannose transporter [12]. As the sole supplier of the substrate required for protein glycosylation in the Golgi, VRG4 is an essential gene [12,13]. In this experimental study, the role of GDP-mannose transporter in *F. graminearum* was examined by analysing the *VRG4* gene. Based on the findings of the gene encoding the GDP mannose transporter in *F. graminearum*, both *FgGmtA* and *FgGmtB* were functionally complementary to yeast mutant strains, Deletion of the *FgGmtA* gene did not result in differences in growth and development, stress, and pathogenicity of *F. graminearum*, whereas *FgGmtA* was transcribed at lower levels. Further studies are needed to investigate whether there is functional redundancy between *FgGmtA* and *FgGmtB*.

*F. graminearum* reproduces both asexually and sexually in the field, and wheat scab (FHB) is caused by both asexual and sexual spores [46,47]. The severity of FHB is often positively correlated with the number of conidia or ascospores [1]. The conidiophores produce conidia by a process of asexual reproduction, and these conidia can rapidly germinate into new hyphae under suitable environmental conditions, thereby spreading and multiplying the fungus [48]. Since ascospores produced by sexual reproduction of *F. graminearum* are the main source of infection, further analysis of the effect of *FgGmtA/B* on ascospore formation ability is necessary [49]. After knocked-out *FgGmtB*, the Δ*FgGmtB* mutant did not have abnormal conidial morphology and the growth rate was slowed, but the number of conidia increased, indicating that *FgGmtB* has a negative regulatory effect, and the expression of genes involved in asexual reproduction was also increased. Δ*FgGmtA&B* had severe defects on plates, but conidia formation in liquid CMC cultures was not affected in any way. The conidial suspension of the mutant was cultured into wheat ears and coleoptiles, etc., and the disease was observed after onset. It was found that Δ*FgGmtB* and Δ*fgGmtA&B* knockout mutants were significantly less pathogenic to wheat coleoptiles, wheat heads, and corn silks. Since cellophane penetration experiments showed no defect in the penetration of the mutants, it was hypothesised that this could be a reduction in fungal pathogenicity due to mycelial growth restriction. In addition, the Δ*FgGmtB* and Δ*FgGmtA&B* knockout mutant strains were severely defective in forming ascus shell formation and, consistent with these results, DON production was significantly reduced in Δ*FgGmtB* and Δ*FgGmtA&B*. In the Δ*FgGmtB* and Δ*FgGmtA&B* mutants, the expression levels of *TRI6*, *TRI101*, etc., key genes for DON toxin synthesis, were significantly reduced. Most gene deletion mutants had reduced toxin synthesis and pathogenicity in previous studies; knockout of *FgGmtB* may reduce DON biosynthesis and virulence by indirectly affecting secondary metabolism [50].

In *A. nidulans*, Gmt-deleted strains had smaller colonies with reduced sporulation and thicker hyphal walls. In *A. fumigatus*, inhibition of GDP-mannose pyrophosphorylase (GMPP), which catalyses the synthesis of GDP-mannose, results in a lethal phenotype including hyphal lysis, cell wall defects, and impaired polarity maintenance [24,51]. The present study shows that the role of GMT in colony morphology and cell wall metabolism is consistent with many other findings in yeast and filamentous fungi, and that *FgGmtB* is involved in cell wall integrity (CWI). The fungal cell wall is the outer and first layer of fungi and is critical for sensing environmental signals. In stress sensitivity experiments, the Δ*FgGmtB* and Δ*FgGmtA&B* mutants showed increased sensitivity to cell wall damaging agents compared to wild-type strain PH-1 and the Δ*FgGmtA* mutant strain. In this study, the results of hyphal growth and virulence experiments showed that the virulence inhibition rate of Δ*FgGmtB* and Δ*FgGmtA&B* mutants was much higher than that of hyphal growth. This suggests that the reduced virulence of Δ*FgGmtB* and Δ*FgGmtA&B* mutants is also related to other factors, but it is reasonable to speculate that *FgGmtB*-regulated CWI is involved in the virulence of *F. graminearum*. Therefore, defective hyphal growth and CWI may be the main factors contributing to the reduced virulence of this fungus.

Salt stress is abiotic stress that is a common and important environmental factor limiting crop germination, growth and productivity [52]. The HOG pathway regulates glycerol levels in most organisms through MAPK Hog1 phosphorylation to maintain osmotic homeostasis [53]. The present study measured glycerol accumulation in the tested strains with or without NaCl and showed that glycerol was reduced in Δ*FgGmtB* and Δ*FgGmtA&B* compared to PH-1 and Δ*FgGmtA*, resulting in resistance to external osmotic stress. Meanwhile, under osmotic conditions, wild-type glycerol accumulates to balance the high external osmotic pressure, and isobacturon and fludioxonil interfere with the osmotic signaling pathway, thereby stimulating glycerol biosynthesis [54]. Similar results were obtained in the present study, with Δ*FgGmtB* and Δ*FgGmtA&B* mutants showing increased resistance to isofluranil and fludioxonil and reduced glycerol content.

In this study, a GmtA/B-GFP fusion fragment with the *GmtA/B* self-promoter was randomly inserted into the Δ*FgGmtA/B* mutant to obtain *GmtA/B* gene-complementary transformants. Complementary transformants returned to wild-type status with respect to growth rate, sporulation rate, DON synthesis, and pathogenicity. However, the subcellular localisation of GMT was not found, possibly because the expression of green fluorescent protein is too low, and some studies have found that the expression of the GMT gene may need to be enhanced under acidic conditions, involving the stress response of the cell wall to acid stress, which needs further verification.

Therefore, this study provisionally confirmed that the GDP-mannose transporter *FgGmtB* is indispensable for mycelial growth, pathogenicity, stress response, *TRI* gene expression, and DON toxin biosynthesis of *F. graminearum* potential targets for virulence-determining primers for antifungal drugs that inhibit the translocation of GDP-mannose into the Golgi lumen, which is required for the biosynthesis of the fungal virulence factor galactomannan. At the same time, the results of this study laid the foundation for a deeper understanding of the molecular mechanism of *F. graminearum* pathogenicity and provided a corresponding theoretical basis for the prevention of wheat scab.

## 5. Conclusions

In conclusion, we identified two GDP-mannose transporters and showed that *FgGmtB*, but not *FgGmtA*, is involved in the regulation of vegetative growth, asexual and sexual reproduction, fungicide sensitivity, pathogenicity, and DON biosynthesis and virulence in *F. graminearum*. The next work will mainly focus on identifying the target genes regulated by *FgGmtB*.

## Figures and Tables

**Table 1:** Conidiation, conidial germination in the PH-1, Δ*FgGmtA*, Δ*FgGmtB,* and Δ*FgGmtA&B* mutants. Values on bars followed by different letters statistically different at *p* < 0.05.

Strain	Conidiation (10^5^ Conidia/mL)	Germination (%)
PH-1	9.01 ± 0.25 b	93.78 ± 0.09 b
Δ*fgGmtA*	8.92 ± 0.17 b	92.34 ± 0.12 b
Δ*FgGmtB*	12.67 ± 0.52 a	95.26 ± 0.05 a
Δ*fgGmtA&B*	8.5 ± 0.39 b	90.54 ± 0.07 c

## Data Availability

Data are contained within the article and Supplementary Materials.

## Supplementary Materials

The following supporting information can be downloaded at: https://www.mdpi.com/article/10.3390/jof10030208/s1. Table S1: PCR Primers used in this study. Figure S1: Structural analysis of FgGmts of *F. graminearum*. Figure S2: Knockout strategy and validation of *FgGmtA* and *FgGmtB*. Figure S3: Determination of the utilization of four carbon sources by *FgGmtA/B* in MM medium. Figure S4: Determination of *FgGmtA/B* sensitivity to osmotic stress and corresponding glycerol concentration.
