# Supplementary material for: FgGmtB Plays an Important Role in Growth, Reproduction, Virulence and Deoxynivalenol Biosynthesis of Fusarium graminearum
Source: J Fungi (Basel). 2024 Mar 11;10(3):208. doi: 10.3390/jof10030208 (PMC10971127; doi:10.3390/jof10030208)
Supplement: Supplementary file 1 [file jof-10-00208-s001.zip › jof-2867915-supplementary.pdf]

Supplementary Table S1. PCR Primers used in this study

Primer names	Sequence（5'-3'）
FgGmtA-1F	CATCATTCACGCTACACTGCC
FgGmtA-2R	TTGACCTCCACTAGCTCCAGCCAAGCCTCGGTTTAGCGGAAGAGAGAG
FgGmtA-3F	GAATAGAGTAGATGCCGACCGCGGGTTCATTTGGTAGAGCCTGGTCC
FgGmtA-4R	ACGAAAGCGAAGCCTATCC
FgGmtA-5F	CGGCTCAAGTCAACTCCAC
FgGmtA-6R	CTTGTTGCGTGTATCGTCAG
FgGmtA-7F	CATGAGAACTGTCTTGGCGC
FgGmtA-8R	ACTCGATGGGTCGTTGACTG
FgGmtB-1F	TTGGTCCTCCCAGTAGTAATCG
FgGmtB-2R	TTGACCTCCACTAGCTCCAGCCAAGCCTTGTAAAAGAGTGGACGAACGG
FgGmtB-3F	GAATAGAGTAGATGCCGACCGCGGGTTGCCAATCTTTCCTCCTAGCC
FgGmtB-4R	GCTGGCGTCAAGGATGTG
FgGmtB-5F	GCATGATCAAGTCCCTTGC
FgGmtB-6R	GACGAAACCAATAAAGATAGCTG
FgGmtB-7F	CCACATGAAAGCAATATCAGG
FgGmtB-8R	GTTAAGTGAACCCATGCGAC
HYG/F	GGCTTGGCTGGAGCTAGTGGAGGTCAA
HY/R	GTATTGACCGATTCCTTGCGGTCCGAA
YG/F	GATGTAGGAGGGCGTGGATATGTCCT
HYG/R	AACCCGCGGTCGGCATCTACTCTATTC
H852	TTCCTCCCTTTATTTCAGATTCAA
H850	ATGTTGGCGACCTCGTATTGG
H855R	GCTGATCTGACCAGTTGC
H855F	GTCGATGCGACGCAATCGT
FgGmtA-NEO/1F	CATCATTCACGCTACACTGCC
FgGmtA-NEO/2R	GTAAGCGTTAATCTAGAGCGTCGGTTTAGCGGAAGAGAGAG
FgGmtA-NEO/3F	TGGGTAAACGACTCATAGGAGCATTTGGTAGAGCCTGGTCC
FgGmtA-NEO/4R	ACGAAAGCGAAGCCTATCC
NEO-1F	CGCTCTAGATTAACGCTTAC
NEO-2R	CCTGATGTTCTTCGTCCA
NEO-3F	GACAATCGGCTGCTCTGA
NEO-4R	CTCCTATGAGTCGTTTACCCA
N852-F	TCGGCTATGACTGGGCACAACA

N852-R	GAGCGGCGATACCGTAAAGCAC
N855-F	GAATGGTCAAATCAAACTGCTAGATAT
N856-R	TTGACTGGCACTGCTGGG
T6-QF	CCCTCAGTCAGCTCAAGACC
T6-QR	CACCCTGCTAAAGACCCTCA
T10-QF	GCTCCCAAATCTTGAAGCTG
T10-QR	AGCGTCTTCCAAGACCTGAA
T12-QF	CATCAGCCTCTGCTCTTCCCA
T12-QR	GTCGCCCAAATCTATCCGTAA
T101-QF	GCCTTCGTTCAGAACTCGAC
T101-QR	CCCAAAGTCGTAATCCCAGA
CHS7-QF	TCGTTATCTCACCACCCTGA
CHS7-QR	AAACACAACGAAGCGCATAC
AbaA-QF	ACTCAGGAAGCTTTGACCACGGC
AbaA-QR	GGGCTCTGGTAGGGGTTGACAGTA
WetA-QF	GTTCCAGGTACTCCCACTGCCAT
WetA-QR	ACGTTCTCGTCGCGCTTTGGT
Mat1-QF	TTCGGTCCTGATTACGATGCC
Mat1-QR	CCAAGCGCTGGTGATCTCCATG
Mat2-QF	AATCACCCAGGCACTCGACGT
Mat2-QR	GCGTGAACGGCTGTGGACAT
mat3-QF	ATCAATCTACTCCAGCGACG
Mat3-QR	ACGCCAGTAAGCCTGTGTCTC
Mat4-QF	AACGCTTACATCCTCTACCG
Mat4-QR	CCTGAGCGCTAGACACGTGAGG
pYES2-FgGmtB-F	GATGCGGCCCTCTAGAATGGCCGACCAGAACAAGAAG
pYES2-FgGmtA-R	TACCGAGCTCGGATCCCTTTGACGAATCTCGGTTCTCG
pYES2-FgGmtA-F	GATGCGGCCCTCTAGAATGACCGATAAGAAGAACGAGG
pYES2-FgGmtA-R	TACCGAGCTCGGATCCAGAGTTGGCATCACGGTTGC
FgGmtA-CF	AGGGAACAAAAGCTGGGTACCGGCGTACTTACGATGTCCAGATC
FgGmtA-CR	GAACAGCTCCTCGCCCTTGCTCACAGAGTTGGCATCACGGTTGC
FgGmtB-CR	AGGGAACAAAAGCTGGGTACCGTTGCCAAAGTTGTCCATGAGC
FgGmtB-CR	GAACAGCTCCTCGCCCTTGCTCACCTTTGACGAATCTCGGTTCTCG

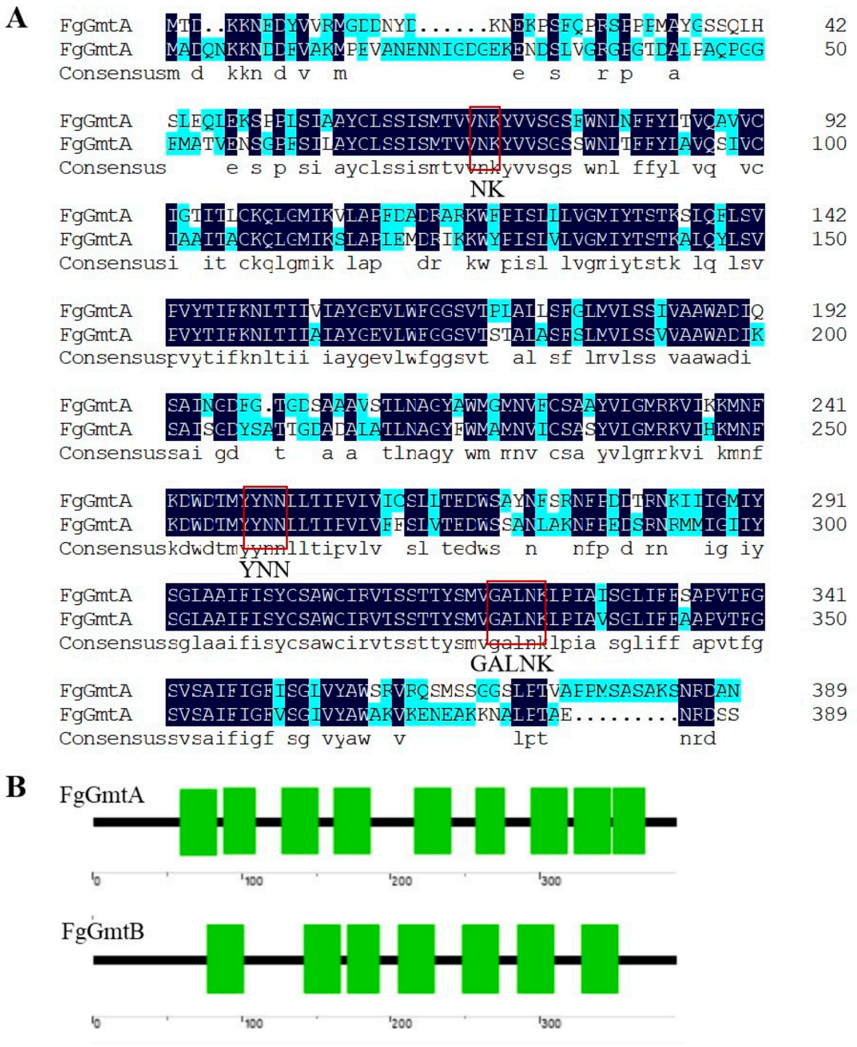

Supplementary Figure S1. Structural analysis of FgGmts of *F. graminearum*. (A) Multiple sequence alignments of *FgGmtA*, and *FgGmtB* were built with DNAMAN 6.0 software (Lynnon Biosoft, Foster City, CA, USA), and three conserved motifs (NK, YNN and GX(L/V)NK) are marked in a box. (B) Transmembrane structure of *FgGmts*.

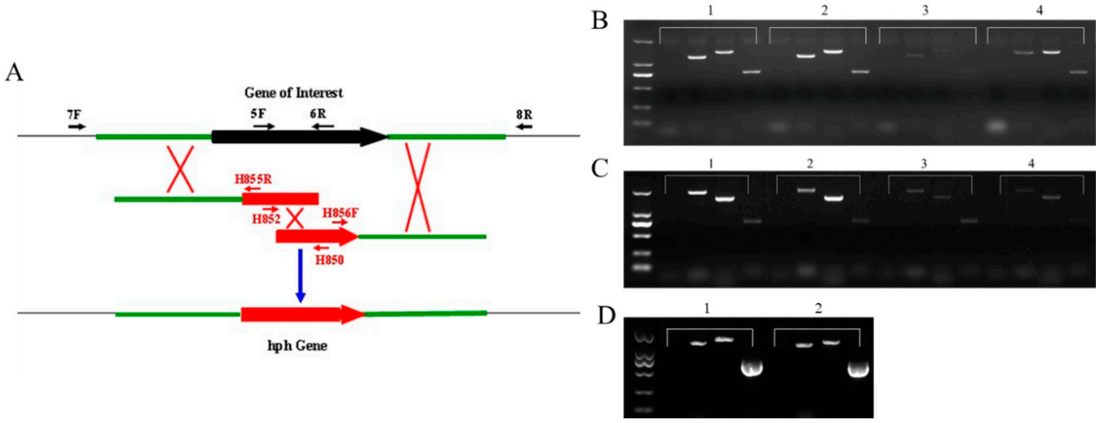

Supplementary Figure S2. Knockout strategy and validation of *FgGmtA* and *FgGmtB*. (A) Schematic of homologous recombination-based gene knockout and four-pair primer PCR detection of transformants. (B) Detection of *FgGmtA* knockout transformants. (C) Detection of *FgGmtB* gene knockout transformants. (D) Detection of *FgGmtA&B* gene knockout transformants.

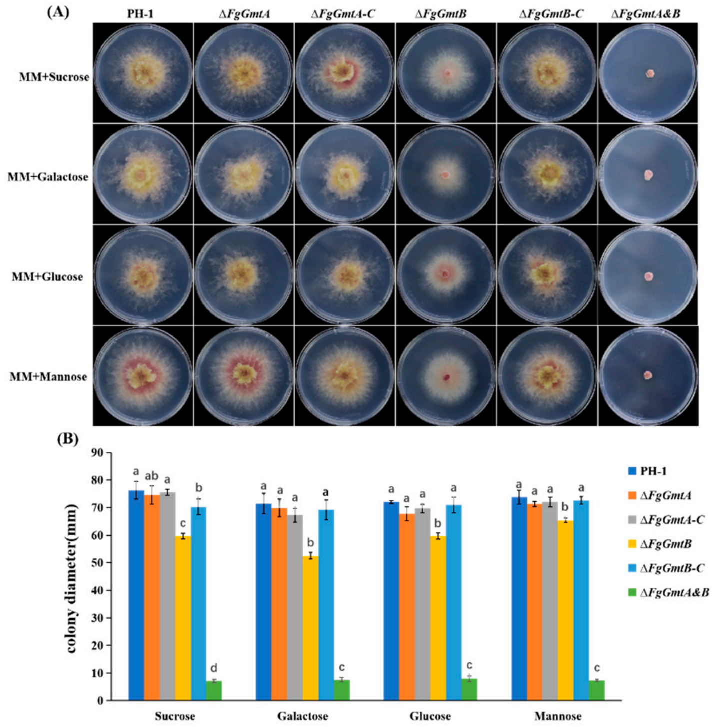

Supplementary Figure S3. Determination of the utilization of four carbon sources by *FgGmtA/B* in MM medium. (A) The growth of wild-type PH-1, *ΔFgGmtA*, *ΔFgGmtB* and *ΔFgGmtA&B* strain complemented strain on MM medium containing different carbon sources. (B) the colony diameter of the strain after 3 days of growth on the MM plate. Values on bars followed by different letters statistically different at $p < 0.05$.

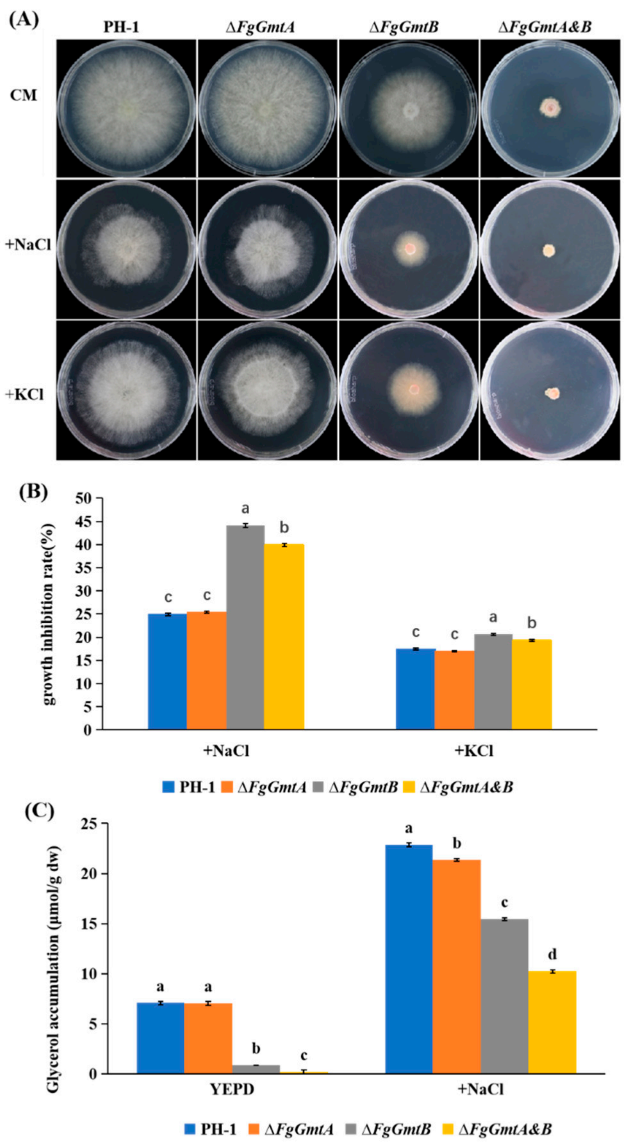

Supplementary Figure S4. Determination of *FgGmtA*/*B* sensitivity to osmotic stress and corresponding glycerol concentration. (A) Colonies of wild-type PH-1, *ΔFgGmtA*, *ΔFgGmtB*, *ΔFgGmtA&B*, PH-1 mutant strains on PDA media supplemented with or without 1.2 mol/L NaCl and 1.2 mol/L KCl. (B) Growth inhibition rate of each strain after incubation at 25 °C for 3 days, calculated from 3 independent experiments. (C) Intracellular glycerol accumulation in cells treated for 2 hours with or without 1.2 mol/L NaCl. Values on bars followed by different letters statistically different at $p < 0.05$.
